# Protective Effects of Some Graded Iranian Honey Samples Against Cold Water Immersion‐Induced Gastric Ulcers in Rats

**DOI:** 10.1002/fsn3.4567

**Published:** 2024-11-01

**Authors:** Yaser Mohammadi, Zoya Tahergorabi, Gholam Reza Sharifzadeh, Mahdieh Rajabi Moghadam, Asghar Zarban

**Affiliations:** ^1^ Department of Clinical Biochemistry, School of Medicine Birjand University of Medical Sciences Birjand Iran; ^2^ Department of Biochemistry, School of Medicine Iran University of Medical Sciences Tehran Iran; ^3^ Geriatric Health Research Center Birjand University of Medical Sciences Birjand Iran; ^4^ Department of Epidemiology and Biostatistics, Social Determinants of Health Research Center, School of Health Birjand University of Medical Sciences Birjand Iran; ^5^ Department of Pathology, Faculty of Medicine Birjand University of Medical Sciences Birjand Iran

**Keywords:** cold water immersion stress, rat, stress ulcer, strong honey, weak honey

## Abstract

Honey has a rich history of treating gastrointestinal diseases due to its diverse bioactive compounds. This study evaluated the protective effects of select Iranian honeys against cold water immersion stress (CWIS)‐induced ulcers in rats. Forty male Wistar rats (250–280 g) were randomly assigned to eight groups (*n* = 5): control, CWIS, and groups treated with strong (eucalyptus, Annaab, and Jangale) and weak honeys (Chand Giah, Sumaq, Gaz) + CWIS. Honey selection was based on antioxidant capacity, phenolic content, and protein concentration. Rats received 20% honey in water (1 mL/kg) orally twice daily for 14 days; controls received water. After a 24‐h fast, rats underwent 3‐h CWIS to induce ulcers. Serum samples were analyzed for *malondialdehyde* (MDA), *total antioxidant capacity* (TAC), thiol groups, tumor necrosis factor alpha (TNF‐α), and *interleukin 6* (IL‐6) levels. Stomachs were assessed for ulcer severity (gastric ulcer index), gastric juice volume, and histopathological changes. Honey types were categorized as strong (eucalyptus, Annaab, and Jangale) or weak (Chand Giah, Sumaq, and Gaz) based on total phenolic content, antioxidant effects (FRAP and DPPH), and protein levels. Ulcer group showed significant increases in MDA (64%) and TNF‐α and IL‐6 levels (98.5% and 111.6%), and decreases in ferric reducing *antioxidant* power and 2,2‐diphenyl‐1 picrylhydrazyl (FRAP, DPPH, and thiol levels [26%, 14.39%, and 26%]) compared to controls. Strong honey groups exhibited 50% lower gastric ulcer index compared to weak honey groups. This study showed that strong honeys, due to their higher phenolic, total protein, and antioxidant content, offer greater protection against gastric damage and oxidative stress compared to weaker honeys. These results highlight the importance of bioactive compounds in honey's therapeutic properties. Therefore, high‐quality honeys with higher phenolic content can be considered as therapeutic supplements for gastrointestinal disorders, especially those caused by oxidative stress and inflammation.

## Graphical abstract

This manuscript represents original research in the field of therapeutic and protective effects of honey. In this study, we observed that honey samples with high phenolic content provide a more robust protective shield against gastric ulcers in an animal model. This valuable finding represents a significant step forward in our understanding of the therapeutic and protective potential of honey.

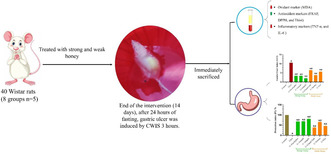

**Abbreviations**
ACTH: adrenocorticotropic hormone
CRH: corticotropin‐releasing hormone
CRS: cold‐restraint stress
CWIS: cold water immersion stress
FBS: fasting blood sugar
FRAP: ferric reducing/antioxidant power
GA: gallic acid
GI: gastrointestinal
GSH: glutathione
GUI: gastric ulcer index
GWM: gastric wall mucus
H&E: hematoxylin and eosin
H2RA: histamine 2 receptor antagonists
HPA: hypothalamic–pituitary–adrenal
ICU: intensive care unit
IL‐1β: interleukin‐1β
IL‐6: interleukin 6
LPO: lipid peroxidation
LPS: lipopolysaccharide
MDA: malondialdehyde
PAS: periodic acid Schiff
PI: protection index
PPI: proton pump inhibitors
ROS: reactive oxygen species
SOD: superoxide dismutase
SRMD: stress‐related mucosal damage
TAC: total antioxidant capacity
TBARs: thiobarbituric acid reactive
TE: Trolox equivalent
TNF‐α: tumor necrosis factor alpha
TPC: total phenolic content
WIS: water immersion stress

## 1 Introduction

Stress, as an integral part of human life and organisms, can affect numerous physiological processes and depending ability to cope with stressful stimuli trigger changes in different organ systems, including the gastrointestinal (GI) system (Metreveli and Japaridze 2022).

Stress ulcer, also known as stress‐related mucosal damage (SRMD), is a broad term describing the spectrum of acute, erosive, and inflammatory injuries that primarily affect the surface epithelium and can extend into deeper, localized, and penetrating submucosal layers, typically impacting the GI system, particularly the fundus and body of the stomach. (Tsige et al. 2023). The hypothalamic–pituitary–adrenal (HPA) axis with a cascade of hormones, including *corticotropin‐releasing hormone* (CRH), adrenocorticotropic hormone (ACTH), and cortisol, is a major part of the neuroendocrine system in exposure to stress that plays a role in stress ulcer formation and development (Karin et al. 2020).

Stress ulcers may lead to major bleeding complications following critical illness such as shock, trauma, postoperation, and severe bacterial infections (Bardou, Quenot, and Barkun 2015). The incidence of stress ulcers varies over time and among different patient populations. Based on endoscopic studies, 75%–100% of critically ill patients suffer from SRMD within 24 h of admission to the intensive care unit (ICU) (Yang and Lewis 2003). Furthermore, according to an international prevalence study by Krag et al. (2015), 7.3% of patients experience GI bleeding during the ICU stay which in turn causes additional hospital costs and increases mortality up to four times.

The precise mechanism of stress ulcers remains unclear; however, the disruption of the balance between aggressive factors (gastric acid and pepsin) and protective mechanisms (mucosal blood flow and prostaglandin E2) is considered the most significant factor in gastric ulcer formation, and stress ulcers are no exception(Alshami et al. 2019). Hemodynamic and inflammatory factors are recognized as the underlying pathophysiology of SRMD, which frequently occurs in critically ill patients (Buendgens, Koch, and Tacke 2016). Reactive oxygen species (ROS) molecules and inflammatory processes contribute to epithelial necrosis and mucosal ulceration. Malondialdehyde (MDA), superoxide dismutase (SOD), and glutathione (GSH) serve as indicators reflecting the severity of tissue oxidative damage (Wang et al. 2020).

Treatment of SRMD remains a major challenge for health professionals although pharmacological stress ulcer prophylaxis with proton pump inhibitors (PPI) or, less frequently, histamine 2 receptor antagonists (H2RA) are routinely used based on recommendations of current guidelines for critical care in ICU around the world; however, the administration of a PPI is certainly not a risk‐free prophylactic intervention (Buendgens and Tacke 2017).

The high incidence and life‐threatening consequences of stress ulcers together with the increasing pattern of antibiotic resistance as an emerging problem in many countries necessitate adoption of various therapeutic strategies aiming at the prevention of stress ulcer disease.Since ancient times, natural products such as honey have been used as a therapeutic agent to promote good digestive health due to antibacterial, antifungal, antiviral, hepatoprotective, antioxidant, and anti‐inflammatory properties also in the healing of wounds, burns, and infectious (Bukhari et al. 2011).

Gastric ulcers induced by cold water–restraint stress (CWRS), cold‐restraint stress (CRS), or water immersion stress (WIS) are widely accepted for studying stress ulcers and mimic acute gastric ulcerations caused by trauma, surgery, or sepsis (Adinortey et al. 2013). Therefore, this study aimed to determine the protective effect of some Iranian honey samples against stress ulcers induced by cold water immersion stress (CWIS) in rats.

## 2 Materials and Methods

### 2.1 Honey Samples

**FIGURE 1:**
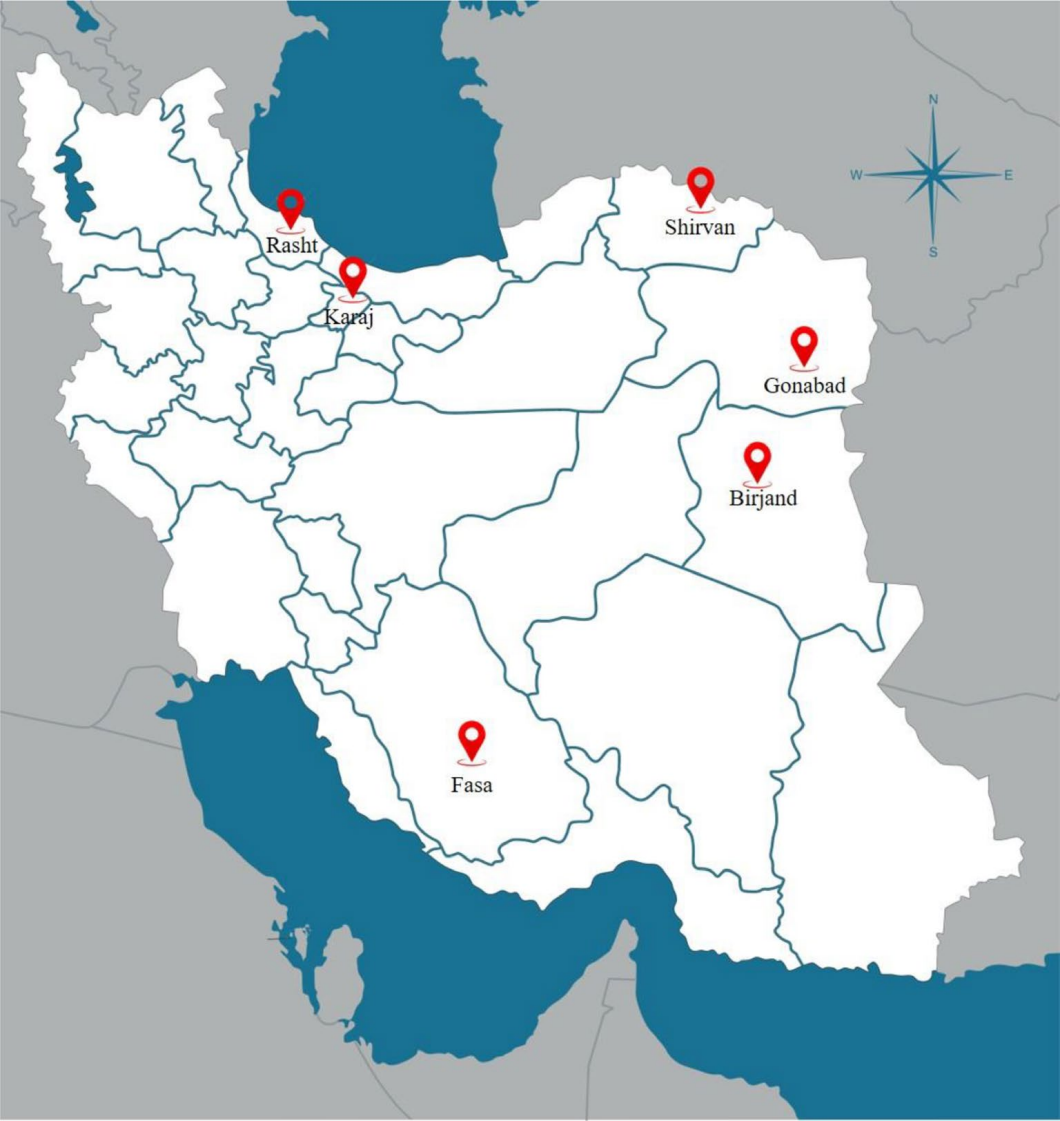
Honeys from different provinces of Iran. These areas include Gonabad, Rasht, Fasa, Karaj, Shirvan, and Birjand.

During the 2022 harvest season, several honey samples were collected directly from beekeepers or markets and were analyzed for total antioxidant capacity (TAC), total phenolic compounds (TPC), and total proteins, which serve as key indicators of the medicinal and therapeutic properties of natural honey samples. Six honey samples from six distinct Iranian regions (Figure 1, Table 1) were selected for analysis. To categorize the samples into strong and weak groups, we evaluated their TPC, total proteins, and antioxidant capacity. Specifically, honey samples exhibiting higher levels of phenolic compounds, total proteins, and greater antioxidant activity were classified as “strong,” while those with lower levels were categorized as “weak.” This classification allowed us to investigate the potential health benefits associated with different types of honey more effectively. The samples were stored at room temperature in a dark environment to preserve their properties.

**TABLE 1:** Classification of honey types and regional sources.

Samples	Common name	Botanical name	Family	Geographical region
H1	Eucalyptus	*Eucalyptus globulus* Labill	Myrtaceae	Fasa/Fars province
H2	Annaab	*Ziziphus jujube* mill	Rhamnaceae	Birjand/South Khorasan province
H3	Jangale	Multifloral		Rasht/Gilan province
H4	Chand Giah	Multifloral		Shirvan/North Khorasan province
H5	Sumaq	*Rhus coriaria*	Anacardiaceae	Gonabad/Razavi Khorasan province
H6	Gaz	*Tamarix* sp.	Tamaricaeae	Karaj/Alborz province

### 2.2 Analysis of Honey Samples

#### 2.2.1 Total Phenolic Content (TPC)

The determination of TPC in the collected honey samples was conducted using the Folin–Ciocalteu reagent method with gallic acid (GA) serving as the standard. The procedure involved diluting 0.1 mL of various GA concentrations (ranging from 0.0078 to 0.5 mg/mL) or methanolic honey solutions (1 mg/mL) with 5.0 mL of distilled water. Subsequently, 0.5 mL of 0.2 N Folin–Ciocalteu reagent was added, and the mixture was vortexed. After a 3‐min incubation, 1.5 mL of 2% Na_2_CO_3_ solution was introduced, followed by vertexing, and the mixture was left to incubate for 2 h at 20°C with intermittent shaking. The absorbance was measured at 760 nm at the conclusion of the incubation period relative to a blank. The concentration of total phenolic compounds was determined as milligrams of GA equivalent per 100 g of dry honey sample, employing a standard graph (Slinkard and Singleton 1977).

#### 2.2.2 Ferric Reducing/Antioxidant Power (FRAP) Assay

The fundamental principle of this method involves the reduction of a ferric 2,4,6‐tripyridyl‐s‐triazine complex (Fe^3+^‐TPTZ) to its ferrous‐colored form (Fe^2+^‐TPTZ) in the presence of antioxidants. The FRAP reagent comprised 2.5 mL of a 10 mM TPTZ solution in 40 mM HCl, 2.5 mL of 20 mM FeCl_3_, and 25 mL of 0.3 M acetate buffer with a pH of 3.6. This reagent was freshly prepared each day and stored in the dark at 37°C. Honey solution aliquots of 100 μL were combined with 3 mL of FRAP reagent, and the absorbance of the resulting mixture was spectrophotometrically measured at 593 nm after a 4‐min incubation at 37°C, using a blank solution as reference. Under the same conditions, Trolox served as the standard antioxidant, and the outcomes were expressed in micromoles of Trolox equivalent (TE) per 100 g of dry honey sample (Erturk, Kalın, and Ayvaz 2019).

2,2‐Diphenyl‐1 picrylhydrazyl (DPPH) assay, widely employed for assessing antioxidant capabilities, involves the use of the stable DPPH radical. In this method, DPPH serves as the free radical. The assay relies on spectrophotometric measurement of concentration changes occurring as DPPH reacts with an antioxidant. In summary, 0.75 mL of honey solution at varying concentrations was combined with 0.75 mL of a 100 μM DPPH solution prepared in methanol. The mixture was vigorously vortexed, left in the dark for 50 min, and the activity value for each sample was computed from absorbance values measured at 517 nm. A blank experiment was conducted to ascertain the absorbance of DPPH without any sample. The antioxidant concentration required to achieve 50% radical scavenging, denoted as SC_50_ (mg/mL), was determined from the curves by plotting activity values (scavenging ratio, percent) for corresponding sample concentrations. This parameter was employed to assess the radical scavenging activities of the samples (Erturk, Kalın, and Ayvaz 2019; Slinkard and Singleton 1977).

#### 2.2.3 Protein Assay

In this method, copper is reduced by proteins. The produced Cu^2+^ ion, the bicinchoninic reagent, forms a colored complex, and the absorbance of the resulting mixture was spectrophotometrically measured at 593 nm.

### 2.3 Animals

Male Wistar rats weighing 250–280 g were obtained from the center of experimental studies and laboratory animals of Birjand University of Medical Sciences. They were housed in cages with a 12‐h light‐and‐dark cycle, maintained at 25°C ± 2°C and 65%–70% relative humidity. Standard rat chow was supplied to the rats and they had unlimited access to water. The Birjand University of Medical Sciences Research Ethics Committee accepted the study with ethical code (IR.BUMS.REC.1402.058).

### 2.4 Experimental Design

In the experimental study, 40 male Wistar rats randomly were divided into eight groups (*n* = 5): control group (rats without any intervention), stress ulcer model group (no treatment but ulcer induction), stress ulcer model + strong honey No. 1, stress ulcer model + strong honey No. 2, and stress ulcer model + strong honey No. 3; and stress ulcer model + weak honey No. 1, stress ulcer model + weak honey No. 2, and stress ulcer model + weak honey No. 3. Following a week of acclimation to the new surroundings, the animals received a 20% honey in dose volumes of 1 mL/kg via oral at two times per day for 14 days (the 20% honey dose is made by dissolving 2 g of honey in 10 mL of water). For stress ulcer induction, animals after 24‐h fasting were immersed up to their xiphoid in cold water (20°C–25°C) for 3‐h CWIS on the final day (Adinortey et al. 2013). Then, the animals in all groups were sacrificed with 50 mg/kg ketamine and 10 mg/kg xylazine intraperitoneally.

### 2.5 Gastric Ulcer Index (GUI) Determination

In order to examine macroscopic of gastric ulcer, the stomachs were opened along their larger curvature, cleaned and rinsed with cold normal saline, blotted dry between sheets of filter paper, and pinned flat on cardboard (Guth, Aures, and Paulsen 1979). The severity of CWIS‐induced gastric ulcers was assessed using a grading system that considers the number, size, and depth of ulcers. The degree of ulceration was graded as follows (Raish et al. 2021): 0 indicates no lesions (normal stomach); 0.5 indicates hyperemia (red coloring); 1, hemorrhagic patches; 2, one to five small ulcers; 3, several small ulcers; 4, numerous small and big ulcers; and 6, a whole stomach filled with perforations and ulcers. The following formula was used to get the protection index (PI):

$$\mathrm{PI}=\frac{\text{ulcer model}-\text{ulcer treated}}{\text{ulcer model}}\times 100$$

### 2.6 Measurement of Gastric Acidity

Initially, the amount of gastric juice was measured after the contents of the stomach were properly collected. After that, it was centrifuged at 1000 g for 10 min. The supernatant was taken out and diluted 1 mL at a ratio of 1:10. 0.01 N NaOH was used for the titration. As an indicator, Topfer's reagent was employed. The amount of NaOH used was determined as total acidity when the solution turned blue, and as free acidity when it turned orange (Nigam and Paarakh 2011).

$$\text{Acidity}\;\left(\mathrm{mEq}/L\right)=\frac{\text{Volume of NaOH}\times \text{Normality of NaOH}\times 100}{0.1}$$

### 2.7 Determination of Gastric Wall Mucus (GWM)

The mucus test for stomach epithelia was performed in accordance with Corne, Morrissey, and Woods (1974) methodology. Each stomach's glandular segments were taken out, weighed, and then quickly placed in 10 mL of 0.1% w/v Alcian blue solution, which included 0.16 mL of sucrose solution buffered with sodium acetate (0.05 mL of pH 5) for a 2‐h immersion. Using 10 mL of 0.25 M sucrose to remove the excess dye from each segment, the remaining dye was washed off for 30 min with 10 mL of 0.5 M magnesium chloride. After removing the remaining tissue, it was combined with 4 mL of ethyl ether, agitated for 2 min, and then centrifuged for 10 min at 3000 g. At 598 nm, the supernatant's absorbance was measured (Saremi et al. 2019).

### 2.8 Measurement of Oxidative Stress Markers

The measurements of TAC, MDA, and thiol levels were conducted in accordance with the guidelines provided by ZANTOX (Kavosh Aryan Azma [KAA], Birjand, Iran) (Mohammadi et al. 2022, 2023). The thiobarbituric acid reactive substance (TBARS) technique was used to quantify MDA. FRAP and DPPH test were used to quantify TAC. The thiol groups were measured using the Ellman technique (Ellman 1959).

### 2.9 Measurements of Inflammatory Cytokines

Serum levels of tumor necrosis factor alpha (TNF‐α) and interleukin‐6 (IL‐6) were measured in accordance with the guidelines provided by commercially available rat enzyme immunoassay kits (R&D Systems Inc., Minneapolis, MN, USA).

### 2.10 Histopathology

After being preserved in a 10% formalin buffer, the stomach tissues were cut into 5 μm slices, covered in paraffin, and stained with both periodic acid Schiff (PAS) stain solution and hematoxylin and eosin (H&E). They were then examined under a light microscope for histological and mucosal evaluations (Raish et al. 2021).

### 2.11 Statistical Analysis

Data were analyzed using central and dispersion indices after entering into SPSS16 software. To test the normality assumption, we used the Shapiro–Wilk test. If the assumption was met, we used one‐way ANOVA and the appropriate post hoc Tukey test to compare groups. If the assumption was not met, we used the nonparametric equivalent of the tests (Wilcoxon–Kruskal–Wallis). The significance level was set to < 0.05 for all tests.

## 3 Results

### 3.1 Biochemical Analysis of Honey Samples

According to the findings of the biochemical examination of the honey samples, eucalyptus, Annaab, and Jangale honeys are classified as strong honeys because they include greater levels of TPC, antioxidant effects (FRAP and DPPH), and protein. Additionally, Chand Giah, Sumaq, and Gaz honeys were classified as weak honeys due to their lower levels of protein, antioxidant properties (FRAP, and DPPH), and TPC (Table 2). Thus, it can be said that strong samples of honey have stronger antioxidant and antimicrobial properties than weak samples. It is clear that the values of antioxidant capacity of honey in the FRAP assay were higher than the DPPH.

**TABLE 2:** Results of several honey samples' biochemical analyses together with a classification of those samples according to those results.

Samples	Name	TPC (mg GA/100 g honey)	FRAP (μM Fe^2+^/100 g honey)	DPPH (μM TE/100 g honey)	Proteins (mg/100 g honey)	Classification
H1	Eucalyptus	206	286	190	201	Strong
H2	Annaab	504	606	286	335	Strong
H3	Jangale	136	230	140	286	Strong
H4	Chand Giah	60	67	31	22	Weak
H5	Sumaq	67	87	37	33	Weak
H6	Gaz	61	94	0	0	Weak

### 3.2 GUI and PI

**FIGURE 2:**
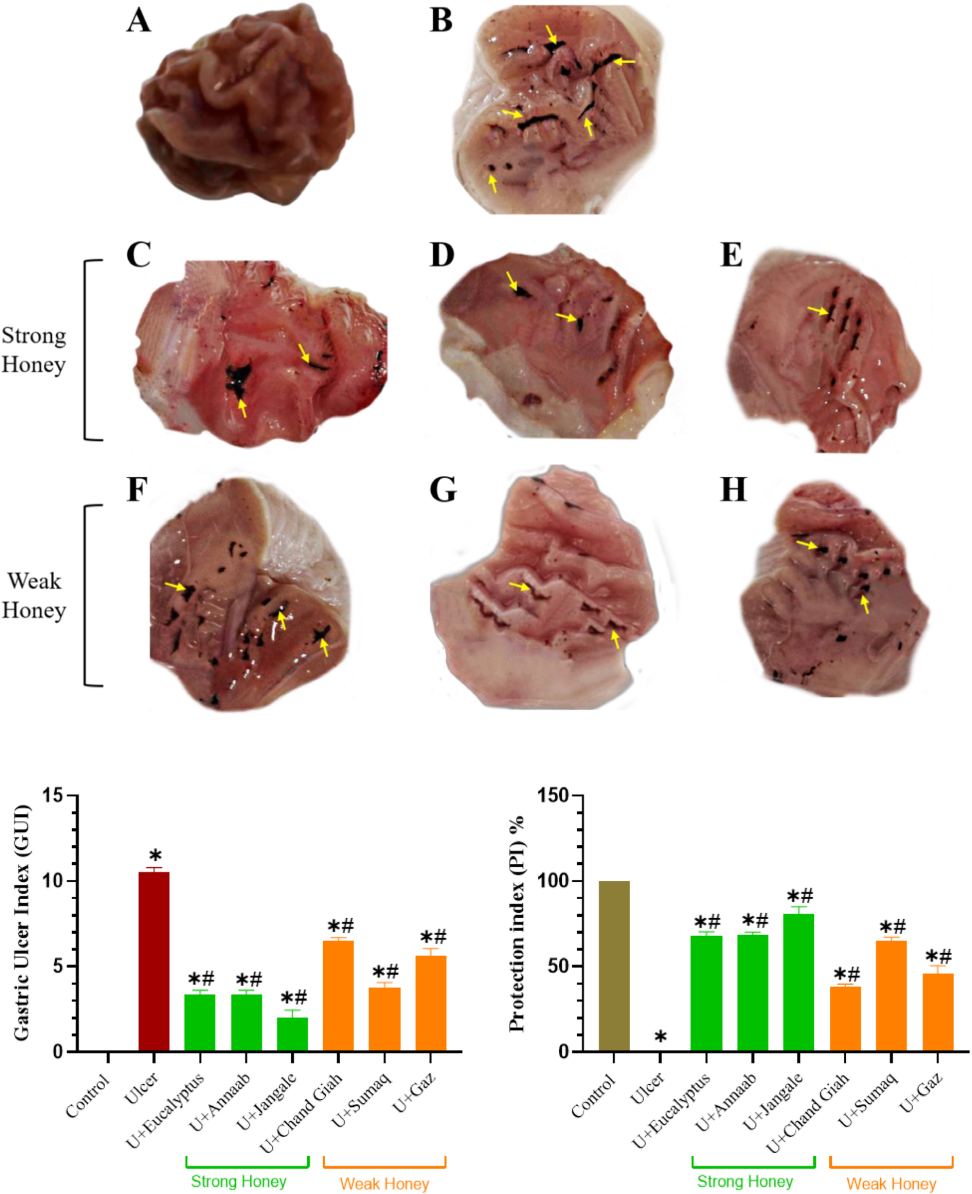
Representative gross views of rat gastric ulcer. (A) The gastric mucosal tissue architecture was normal in the control group. (B) Rats in the ulcer group that were not treated had significant and widespread hemorrhage, with a GUI of 10.5 and a PI of 0%. (C) Rats that received eucalyptus honey had PI 67.7 ± 2.6% and GUI 3.37 ± 0.16, as well as less hemorrhagic lesions of the gastric mucosa than ulcer control animals. (D) In comparison to the ulcer group, rats treated with Annaab honey had GUI ulcers 3.3 ± 0.2 and PI 68.2 ± 1.9%, as well as less hemorrhagic lesions of the gastric mucosa. (E) The Jangale honey treated rats exhibited mild hemorrhagic lesions of the gastric mucosa compared with ulcer model animals, with a GUI of 2 ± 0.4 and a PI of 80.9 ± 4.1%. (F) In comparison to the ulcer model group, the rats that received Chand Giah honey had less hemorrhagic lesions, with a GUI of 6.5 ± 0.4 and a PI of 38 ± 1.7%. (G) Rats that received Sumaq honey showed GUI 3.7 ± 0.3 and PI 64.7 ± 2.5% compared to the ulcer group and the bleeding lesions were somewhat reduced. (H) Compared to the ulcer group, rats that received Gaz honey showed somewhat reduction in hemorrhagic lesions and GUI 5.6 ± 0.4 and PI 45.5 ± 4.9%. ANOVA Tukey test was performed to compare the groups. Data are presented as Mean ± SEM. The yellow arrow indicates gastric ulcer. *Significant difference with the control group (*p* < 0.05). ^#^Significant difference with the ulcer model group (*p* < 0.05).

There was severe and extensive ulcer damage, and clear visible hemorrhagic lesions of gastric mucosal in the stress ulcer model (CWIS) groups compared with control group. Animals treated with various varieties of honey demonstrated a substantial decrease in bleeding lesions or ulcers when compared to stress ulcer model (CWIS) group. The strong honey treatment group showed mean 50% lower GUI compared to the weak honey treatment group. Additionally, the strong honey treatment group had a greater PI and a better level of protection compared to the weak honey treatment group (Figure 2).

### 3.3 Effect of Different Types of Honey on Gastric Mucus and Gastric Juice Acidity

Ulcer model group showed significant higher juice volume, free acidity, and total acidity, as well as significant lower pH and gastric mucus (147%, 239%, 136.60%, and 31%, respectively) in comparison to control group. In contrast to the ulcer model group, strong honey treatment and weak honey treatment groups had significantly lower free acidity, total acidity, and enhanced pH as well as gastric mucus in their stomach. The study's findings demonstrated that strong honey performed better than weak honey because it exhibited a 30% increase in pH, a 30% decrease in free acidity, and a 40% drop in total acidity (Table 3).

**TABLE 3:** Effect of different types of honey on the pH, gastric juice volume, free gastric acidity, total gastric acidity, and gastric mucus content.

Classification of honeys	Groups	pH, mean ± SEM	Gastric juice volume (mL), mean ± SEM	Gastric juice free acidity (mM), mean ± SEM	Gastric juice total acidity (mM), mean ± SEM	Gastric mucus (mg/g tissue), mean ± SEM
	Control	2.5 ± 0.17	1.7 ± 0.14	0.96 ± 0.08	18.3 ± 0.72	7 ± 0.2
	Ulcer	1.5 ± 0.05*	2.5 ± 0.23*	2.3 ± 0.15*	25.2 ± 0.67*	2.2 ± 0.14*
Strong honey	U + Eucalyptus	2.5 ± 0.1^#^	2.7 ± 0.05	1.3 ± 0.06^#^	21.7 ± 0.36* ^,^ ^#^	4.1 ± 0.16 * ^,^ ^#^
	U + Annaab	2.1 ± 0.13^#^	1.5 ± 0.08	1.4 ± 0.06 * ^,^ ^#^	22.6 ± 0.52*	4.8 ± 0.17* ^,^ ^#^
	U + Jangale	2.6 ± 0.13	2.3 ± 0.08	1.5 ± 0.04* ^,^ ^#^	21.3 ± 0.37* ^,^ ^#^	3.2 ± 0.1* ^,^ ^#^
Weak honey	U + Chand Giah	2.1 ± 0.12	1.6 ± 0.12	1.9 ± 0.08*	20.6 ± 0.55^#^	3.3 ± 0.12* ^,^ ^#^
	U + Sumaq	2.7 ± 0.17	2.9 ± 0.21	1.5 ± 0.06* ^,^ ^#^	21.2 ± 0.62^#^	3.9 ± 0.09* ^,^ ^#^
	U + Gaz	2 ± 0.2	2.1 ± 0.14	1.8 ± 0.1* ^,^ ^#^	22.8 ± 0.81*	3.2 ± 0.27* ^,^ ^#^

* Significant difference (*p* < 0.05) when compared to the control group.

^#^ Significant difference (*p* < 0.05) when compared to the ulcer model group. Each group (*n* = 5).

### 3.4 Effect of Different Types of Honey on Inflammatory Factors

**FIGURE 3:**
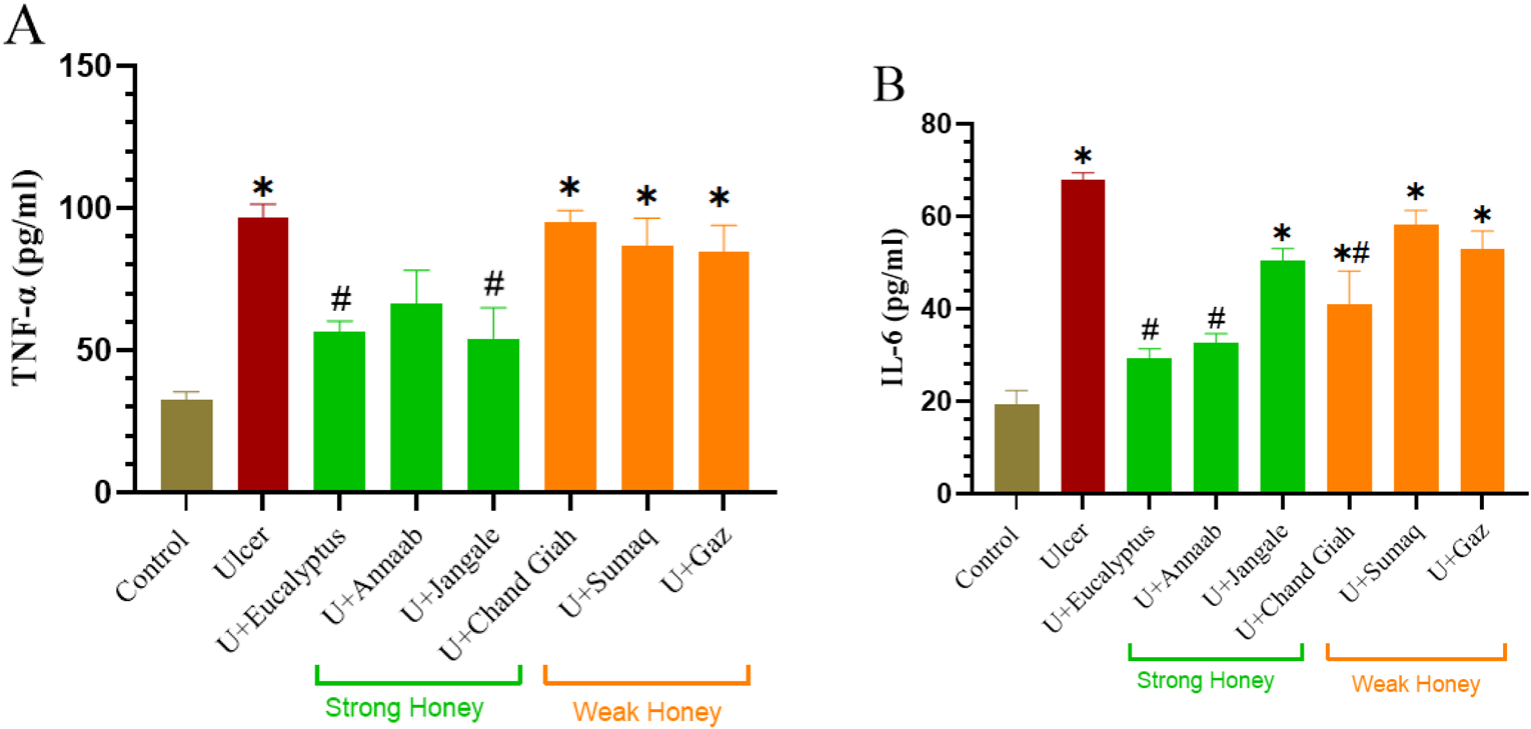
Serum levels of (A) TNF‐α and (B) IL‐6 in strong honey treatment and weak honey treatment groups compared to control groups. Data are presented as the mean ± SEM (*n* = 5) per group. *Significant differences compared with the control group (*p* < 0.05); ^#^significant differences compared with the ulcer model group.

Ulcer model group had significantly higher blood levels of TNF‐α and IL‐6 (98.5%, *p* < 0.05 and 111.6%, *p* < 0.05, respectively) than control group. TNF‐α and IL‐6 levels dropped in the treated groups as compared to the ulcer group; these changes were significant for TNF‐α in the Jangale (56.9%) and eucalyptus (52.39%) groups, and for IL‐6 in the Chand Giah (30.37), Annaab (70.22), and eucalyptus (79.2%) groups. Furthermore, our findings demonstrated that strong honeys had a greater anti‐inflammatory impact than weak honeys (Figure 3).

### 3.5 Effect of Different Types of Honey on Oxidative Stress Markers

The level of MDA, a marker of lipid peroxidation, was significantly increased (64%) in the ulcer model group compared to the control group, while the antioxidant indicators FRAP, DPPH, and Thiol significantly decreased (26%, 14.39%, and 26% respectively). In the treated groups, MDA levels decreased and FRAP, DPPH, and Thiol increased, relative to the ulcer model group; however, these differences were not statistically significant. Overall, our findings demonstrated that strong honeys act better in improving oxidative stress markers compared to weak honeys. As a consequence, in contrast to the ulcer group, the levels of FRAP, DPPH, and Thiol rose by 2.45%, 5.3%, and 7.45%, respectively, and the serum levels of MDA decreased by 30% (Table 4).

**TABLE 4:** Effect of different types of honey on oxidative stress markers (oxidant: MDA and antioxidant: FRAP, DPPH, and thiol group).

Classification of honeys	Groups	MDA, mean ± SEM (μmol/L)	FRAP, mean ± SEM (μmol/L)	DPPH, mean ± SEM (μmol/L)	Thiol, mean ± SEM (μmol/L)
	Control	2.16 ± 0.08	190.3 ± 13.32	205.6 ± 6.17	313 ± 28.8
	Ulcer	3.56 ± 0.36*	145.4 ± 1.85*	178 ± 5.13	240.3 ± 8.83*
Strong honey	U + Eucalyptus	2.65 ± 0.22	153.7 ± 2.95*	187.2 ± 7.2	272.2 ± 15.53
	U + Annaab	2.67 ± 0.11	146.5 ± 4.17*	189.4 ± 5.45	252 ± 14.04
	U + Jangale	2.57 ± 0.18	147 ± 5.04*	187.5 ± 12.3	252.8 ± 12.3
Weak honey	U + Chand Giah	3.02 ± 0.08	161 ± 1.91*	183.2 ± 9.7	256.7 ± 13
	U + Sumaq	2.82 ± 0.28	155.2 ± 5.12*	186 ± 1.86	245.5 ± 6.25*
	U + Gaz	2.67 ± 0.3	163.7 ± 3.49*	183.7 ± 10.7	245.8 ± 2.89

* Significant differences compared with control group (*p* < 0.05).

### 3.6 Effect of Different Types of Honey on Fasting Blood Sugar (FBS) and Body Weight

Our results demonstrated that FBS levels had no significant difference among all groups. The body weight of rats increased as well, although it was not in a way that was statistically significant. The groups that gained the greatest and the least weight were Chand Giah and Gaz, respectively. Changes in FBS level and body weight are shown in Tables 5 and 6.

**TABLE 5:** FBS‐level changes on Days 0, 7, and 14 in the studied groups (data are presented as the mean ± SEM, *n* = 5).

Classification of honeys	Groups	FBS (mg/dL)		
		0‐Day	7‐Day	14‐Day
	Control	90.66 ± 4.8	87.66 ± 2.1	83.78 ± 5.5
	Ulcer	78.67 ± 2.3	84 ± 2.3	87.33 ± 2.3
Strong honey	U + Eucalyptus	85 ± 5	82.25 ± 2	80.22 ± 2.2
	U + Annaab	84 ± 5.5	83.5 ± 4.4	83.45 ± 4
	U + Jangale	87.5 ± 5.3	86 ± 0.9	77.25 ± 4.3
Weak honey	U + Chand Giah	93.75 ± 2.4	91 ± 1.8	84 ± 2.5
	U + Sumaq	91.5 ± 1.2	87.25 ± 3.4	86.65 ± 2.1
	U + Gaz	90.5 ± 1.9	83 ± 3.1	80.75 ± 1.7

**TABLE 6:** Body weight changes on Days 0, 7, and 14 in the studied groups (data are presented as the mean ± SEM, *n* = 5).

Classification of honeys	Groups	Weight body (g)		
		0‐Day	7‐Day	14‐Day
	Control	260 ± 2.8	262 ± 2.9	265 ± 3.1
	Ulcer	263 ± 6	264 ± 5.9	270 ± 7.6
Strong honey	U + Eucalyptus	271 ± 5.5	272 ± 5.3	274 ± 5.1
	U + Annaab	262 ± 7.7	264 ± 8	267 ± 6.3
	U + Jangale	265 ± 3.5	268 ± 3.6	271 ± 3.2
Weak honey	U + Chand Giah	270 ± 5.5	273 ± 5.3	276 ± 5.6
	U + Sumaq	266 ± 5.5	269 ± 5.4	272 ± 5.1
	U + Gaz	263 ± 4.2	264 ± 4.6	268 ± 4.6

### 3.7 Effect of Different Types of Honey on Histopathology of Gastric Tissue

H&E‐staining histopathological examination of the gastric tissue of the ulcer group demonstrated severe mucosal hyperplasia, pits/foveolar decrease, and moderate inflammation. Conversely, control group's gastric tissue showed normal mucosal hyperplasia, no pits/foveolar, and no inflammation. Compared to the ulcer group, the groups treated with strong and weak honey showed less mucosal hyperplasia, pits/foveolar reduction, and inflammation (Table 7).

**TABLE 7:** Effect of different types of honey on histopathology of stomach tissue.

Classification of honeys	Groups	Mucosal hyperplasia	Pits/foveolar decrease	Inflammation
	Control	Normal	Normal	Normal
	Ulcer	Severe	Severe	Moderate
Strong honey	U + Eucalyptus	Normal	Normal	Minimal
	U + Annaab	Normal	Minimal	Minimal
	U + Jangale	Minimal	Normal	Minimal
Weak honey	U + Chand Giah	Mild	Minimal	Mild
	U + Sumaq	Mild	Mild	Mild
	U + Gaz	Minimal	Minimal	Minimal

## 4 Discussion

### 4.1 Main Findings

In general, the therapeutic properties of honey as a natural product are attributed to its bioactive components, which include antibacterial and antioxidant agents. The antioxidant activity of honey is primarily due to its polyphenolic compounds (such as phenolic acids and flavonoids), vitamin C, vitamin E, enzymes (e.g., catalase and peroxidase), and trace elements. These components interact synergistically, enhancing each other's effects and contributing to honey's status as a valuable natural food product (Wieczorek et al. 2014).

Our results indicated that TPC of honeys was 61–504 GAE/100 g, which is in line with Alzahrani et al.'s (2012) study on honeys produced in Germany with TPC ranging 503.09–627.56 mg GAE/100 g and in contrast with Kavanagh et al.'s (2019) study in multifloral Irish honeys TPC ranging from 2.59 to 81.10 mg GAE/100 g.These variations may be attributed to the botanical origin of the honey and the geographical conditions in which the plants grow, both of which influence the TPC of honey (Vîjan et al. 2023).

Our study indicated that the strongest reducing antioxidant power, measured by the FRAP assay, was 606 μmol TE/100 g, while the lowest reducing antioxidant power was 67 μmol TE/100 g. These results align with de Almeida et al.'s study on 15 honey samples from northeast Brazil, where FRAP values ranged from 99.4 to 720.4 μmol TEAC/100 g honey. (de Almeida et al. 2016) In contrast, Chua et al. reported that the reducing antioxidant power measured by FRAP for Gelam honey was 82.529 μmol TE/100 g, while Tualang honey showed the lowest reducing antioxidant power at 52.386 μmol TE/100 g. (2013) Since there is no standardized method for assessing the antioxidant activity of honey, our study employed two established methods: the FRAP, and the DPPH. Notably, the antioxidant capacity of honey was higher in the FRAP assay compared to the DPPH assay. The differences in antioxidant activity among honey samples collected from various regions can be attributed to factors such as botanical origin, climatic conditions, and methods of processing and handling, etc. (Lazarević, Jovetić, and Tešić 2017).

The mean GUI was 50% lower for the strong honey treatment group than the group treated with weak honey, which is in line with Tawfik, Elkady, and Salama (2022) study that showed pretreatment with eucalyptus oil had a potent gastroprotective effect against gamma‐rays–induced gastric ulcer in rats and was associated with ameliorated oxidative stress and increased GSH and SOD, anti‐inflammatory effects (TNF‐α), and the interleukin‐1β (IL‐1β). Also in another study by Nasuti et al. (2006), drug formulations Alimento Supervis and Alimento Mieleucalipto, derived from chestnut honey supplemented with ginseng, propolis, royal jelly, propolis, and eucalyptus, prevented indomethacin‐induced gastric lesions in rats by reducing ulcer index and myeloperoxidase activity of the stomach. Various studies have demonstrated that CWIS due to quick and easy penetration in the stomach membrane causes oxidative effects and lipid peroxidation as increase in MDA and a decrease in the quantity of endogenous antioxidants in the tissues by raising ROS and lipid peroxidation (LPO) and weaken antioxidant defense systems (Suryasa, Rodríguez‐Gámez, and Koldoris 2021).

On the other hand, it has been demonstrated that CWIS leads to nuclear factor kappa B (NF‐ΚB) pathway activation and increases TNF‐α and IL‐1β serum levels. NF‐κB is a transcription factor that is expressed in many systems to play a critical role in inflammatory processes by modulating inflammatory mediators including cytokines, chemokines, and adhesion molecules. Also, various molecules including TNF‐α, lipopolysaccharide (LPS), interleukin 1 (IL‐1), and ROS can lead to activation and induction of NF‐κB (Zhang et al. 2018). Thus, parallel variations in inflammatory factors (TNF‐α and IL‐6) and oxidative stress markers are explained.

Furthermore, rats that received strong and weak honey compared with ulcer group had significantly lower free acidity, total acidity, and enhanced pH, as well as gastric mucus in their stomach, which is in line with de Barros et al.'s (2007) study on green propolis crude extract (250 and 500 mg/kg) that had antisecretory potency and enhanced gastric mucosal defensive factors in stress‐induced ulcer in animal model. Since increase in histamine‐mediated gastric acid secretion and mucus production reduction contributes to development of WIRS‐induced gastric mucosal lesions in rats (Adinortey et al. 2013), possibly in our study, honey(s) in different degrees (strong–weak) possess antisecretory potency and antihistaminic activity.

Macroscopic results and histopathological examination of gastric tissue showed that stronger honeys exhibit greater protective power against ulcers compared to weaker honeys, which is consistent with previous studies. (Das et al. 2022) This may be attributed to their higher phenolic content and greater antioxidant effects. (de Barros et al. 2007)

levels in rats throughout the study, consistent with the findings of Almasaudi et al., who investigated four groups of rats: control, ethanol (ulcer), omeprazole, and manuka honey, and found that honey supplementation did not affect serum glucose concentrations. (Almasaudi et al. 2016) Additionally, eucalyptus has been shown to possess antihyperglycemic activity attributed to its high manganese chloride content (Ismail 2007), and it may also modulate insulin secretion and/or enhance insulin action (Gray and Flatt 1998).

Furthermore, body weight of rats grew as well, although was not in a way that was statistically significant which is in line with Ichi et al.'s (2009) study that 8‐week feeding with pellets containing 0.5% Brazilian propolis did not affect body weight gain in rats. Since obesity and weight gain are closely linked with oxidative stress and inflammation, honey due to its polyphenol and flavonoid content possesses antioxidant and anti‐inflammatory properties (via inhibition of NF‐κB, the key transcription factor of inflammation), therefore it can exert antiobesity actions. As mentioned earlier, factors such as climate, geographic factors, and floral abundance can affect phenolic acid and flavonoid profiles of honey (Cheung et al. 2019).

### 4.2 Strengths and Limitations

Our study has several important strengths. To our knowledge, this is the first research to propose a new classification system for honey based on three key criteria: phenolic compounds, total protein, and antioxidant properties. This system helps to better understand the qualitative differences between various types of honey. Additionally, according to existing knowledge, this study is the first to examine honey from both a dietary and medicinal perspective. The results show that strong honeys, due to their bioactive compounds, exhibit significant medicinal effects, while weaker honeys do not. Another strength of this study is its precise design to evaluate the protective effects of strong and weak honeys against gastric damage caused by oxidative stress and inflammation. Furthermore, the focus on the differences in phenolic content and antioxidant power of honeys and their impact on therapeutic outcomes enhances our understanding of the role of bioactive compounds in protecting the GI system.

However, despite our efforts to conduct a comprehensive study, this research has certain limitations. One major limitation is that the honey samples were restricted to six specific regions of Iran, which may not cover the full geographic and botanical diversity of the country. Additionally, further investigations into the long‐term effects of honey consumption on various health parameters are necessary to fully understand its benefits and drawbacks. Moreover, this study was conducted only on animal models (rats), and the results may not be entirely generalizable to humans, necessitating further research.

## 5 Conclusion

The results of this study demonstrated that strong honeys, due to their higher content of phenolic compounds, proteins, and stronger antioxidant effects, have greater protective effects against cold‐induced gastric damage and oxidative stress. These honeys were able to effectively reduce oxidative stress markers such as MDA and inflammatory activities, including TNF‐α and IL‐6. Additionally, a significant increase in gastric protective mucus and an improvement in acidity levels were observed in the groups receiving strong honey. These effects may be attributed to the strong honeys' ability to neutralize free radicals and enhance the body's endogenous antioxidant defenses. In contrast, weaker honeys, with lower phenolic and antioxidant content, exhibited less protective effects compared to the stronger honeys. This difference clearly highlights the importance of bioactive compounds in enhancing the therapeutic properties of honey. Based on these findings, honeys with higher phenolic and antioxidant content can be considered to be more effective options in preventing and treating GI disorders, particularly those related to oxidative stress and gastric inflammation. Therefore, targeted use of high‐quality honey, based on its bioactive compound content, can lead to improved therapeutic outcomes. Further studies on high‐quality honeys are thus warranted.

## Author Contributions

**Yaser Mohammadi:** conceptualization (equal), data curation (equal), formal analysis (equal), investigation (equal), methodology (equal), writing – original draft (equal), writing – review and editing (equal). **Zoya Tahergorabi:** investigation (equal), methodology (equal), writing – original draft (equal), writing – review and editing (equal). **Gholam Reza Sharifzadeh:** formal analysis (equal), methodology (equal), writing – review and editing (equal). **Mahdieh Rajabi Moghadam:** investigation (equal), methodology (equal), writing – review and editing (equal). **Asghar Zarban:** conceptualization (equal), data curation (equal), methodology (equal), project administration (equal), supervision (equal), writing – original draft (equal), writing – review and editing (equal).

## Conflicts of Interest

The authors declare no conflicts of interest.

## Data Availability

The authors confirm that the data supporting the findings of this study are available within the article.
